# Supplementary material for: Outcomes of patients with malignant duodenal obstruction after receiving self-expandable metallic stents: A single center experience
Source: PLoS One. 2022 May 25;17(5):e0268920. doi: 10.1371/journal.pone.0268920 (PMC9132295; doi:10.1371/journal.pone.0268920)
Supplement: S1 Table — (DOCX) [file pone.0268920.s001.docx]

**S1 Table.** Description of the patients’ levels of oral intake according to the GOOSS score

| GOOSS score | Level of oral intake | Description |
| --- | --- | --- |
| 0 | No oral intake | Patient cannot digest any type of food and water. |
| 1 | Liquid diet only | Patient can digest food with liquid form (e.g., milk, soup, juice). |
| 2 | Soft solids | Patient cand digest food that are soft and easy to chew and swallow (e.g., tender rice or noodles, bread, soft, well-cooked vegetables). |
| 3 | Low-residual or full diet | Patient can digest solid food with low fiber (e.g., rice, noodles, breads, egg) or can digest any type of food. |

GOOSS, Gastric Outlet Obstruction Scoring System
