# Supplementary material for: Outcomes of patients with malignant duodenal obstruction after receiving self-expandable metallic stents: A single center experience
Source: PLoS One. 2022 May 25;17(5):e0268920. doi: 10.1371/journal.pone.0268920 (PMC9132295; doi:10.1371/journal.pone.0268920)
Supplement: S2 Table — (DOCX) [file pone.0268920.s002.docx]

**S2 Table.** Univariate analysis of stent restenosis

| Variables | Univariate analysis | | |
| --- | --- | --- | --- |
|  | *n* | Restenosis, *n* (%) | *p* value |
| Age (≥ 70 / < 70 years old) | 31/46 | 4/13 (12.9/28.3) | 0.324 |
| Sex (male/female) | 43/34 | 10/7 (23.3/20.6) | 0.516 |
| ECOG score (≥ 3/ < 3) | 20/57 | 4/13 (20.0/22.8) | 0.372 |
| Tumor origin  (Pancreatic cancer/ non-pancreatic cancer) | 61/16 | 15/2 (24.6/12.5) | 0.324 |
| Stage (IV/III) | 63/14 | 15/2 (23.8/14.3) | 0.703 |
| Location of obstruction  (Papilla not involved/ Papilla involved) | 44/33 | 9/8 (20.5/24.2) | 0.175 |
| Length of stenosis (≥ 4 / < 4 cm) | 46/31 | 8/9 (17.4/29.0) | 0.593 |
| Length of stent (≥ 9 / < 9 cm) | 56/21 | 12/5 (21.4/23.8) | 0.715 |
| Prior RT (yes/no) | 14/63 | 1/16 (7.1/25.4) | 0.207 |
| Post-stent RT (yes/no) | 9/68 | 2/15 (22.2/22.1) | 0.811 |
| Prior C/T (yes/no) | 38/39 | 6/11 (15.8/28.2) | 0.806 |
| Post-stent C/T (yes/no) | 44/33 | 13/4 (29.5/12.1) | 0.774 |
| Biliary drainage (yes/no) | 47/30 | 12/5 (25.5/16.7) | 0.155 |
| Peritoneal carcinomatosis (yes/no) | 30/47 | 6/11 (20.0/23.4) | 0.987 |
| Liver metastasis (yes/no) | 34/43 | 9/8 (26.5/18.6) | 0.188 |

ECOG, Eastern Cooperative Oncology Group; RT, radiotherapy; C/T, chemotherapy
