# Supplementary material for: Outcomes of patients with malignant duodenal obstruction after receiving self-expandable metallic stents: A single center experience
Source: PLoS One. 2022 May 25;17(5):e0268920. doi: 10.1371/journal.pone.0268920 (PMC9132295; doi:10.1371/journal.pone.0268920)
Supplement: S3 Table — (DOCX) [file pone.0268920.s003.docx]

**S3 Table.** Median survival in patients with and without post-stent chemotherapy

| Type of tumor | Post-stent C/T (-) | | Post-stent C/T (+) | | *p-*value |
| --- | --- | --- | --- | --- | --- |
|  | *n* | Median survival (day) | *n* | Median survival (day) |  |
| All patients | 33 | 71 (10-470) | 44 | 142 (21-887) | <0.001 |
| Pancreatic cancer | 24 | 65 (11-286) | 37 | 161 (21-713) | <0.001 |
| Non-pancreatic cancer | 9 | 71 (10-470) | 7 | 142 (26-887) | 0.336 |

C/T, chemotherapy
