# Supplementary figures and images for: Outcomes of patients with malignant duodenal obstruction after receiving self-expandable metallic stents: A single center experience
Source: PLoS One. 2022 May 25;17(5):e0268920. doi: 10.1371/journal.pone.0268920 (PMC9132295; doi:10.1371/journal.pone.0268920)

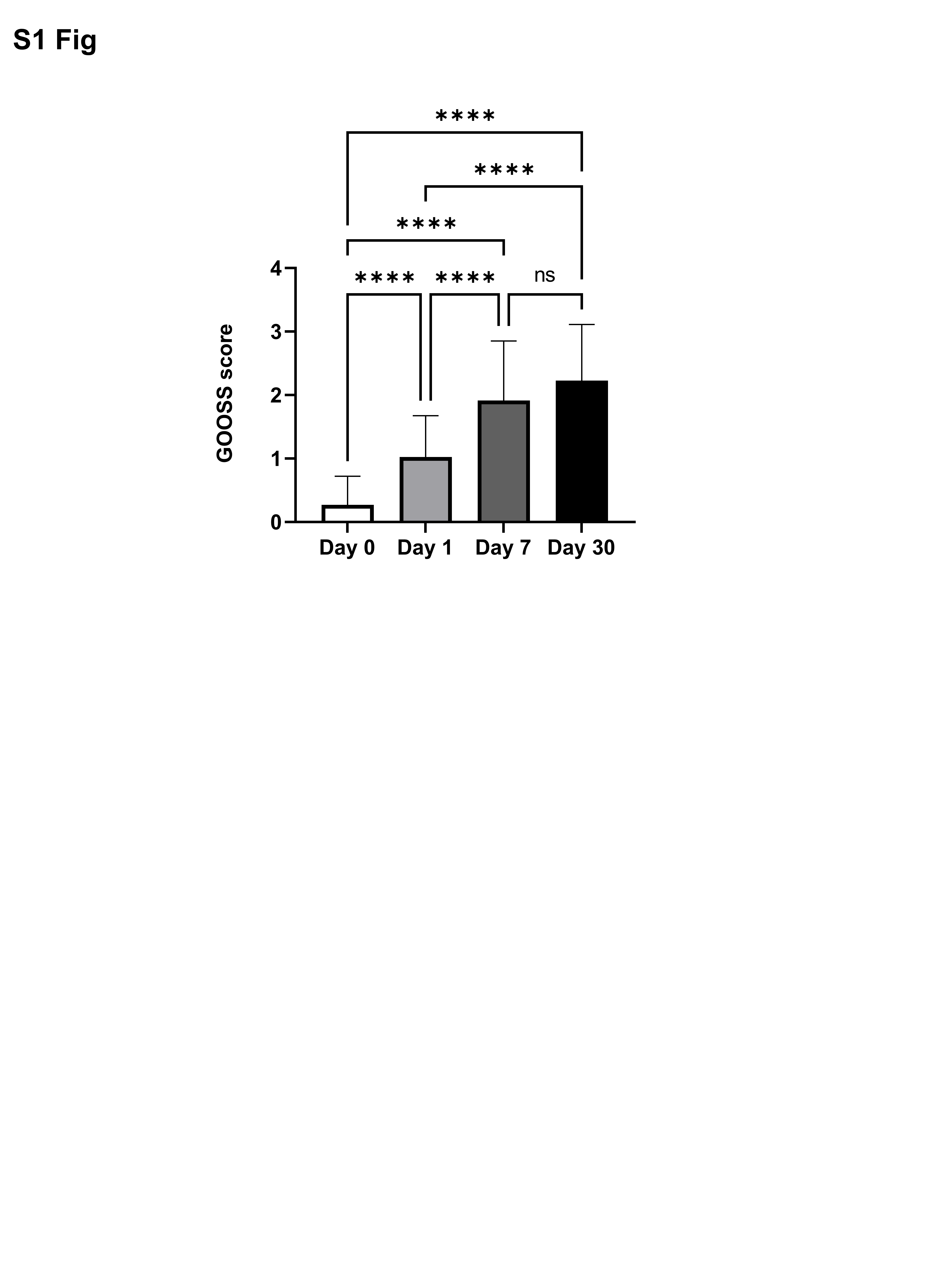

Supplement: S1 Fig — ns, not significant ****: p <0.0001 between the groups determined by Kruskal Wallis test. (TIF) [file pone.0268920.s004.TIF]

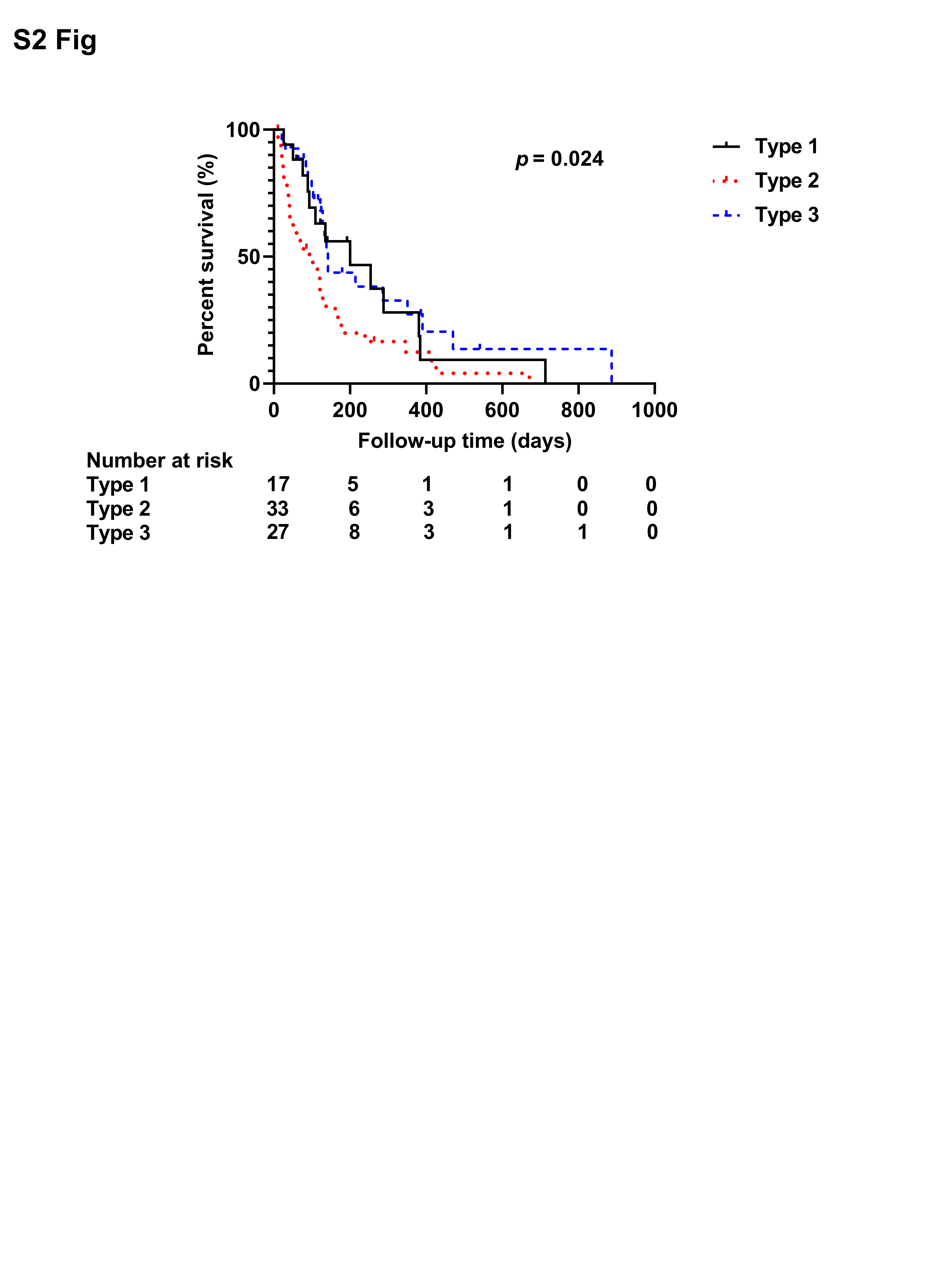

Supplement: S2 Fig — Type 1: the above-papilla group; type 2: the papilla involved group; type 3: the below-papilla group (type 3). The p-value corresponds to log–rank test. (TIF) [file pone.0268920.s005.TIF]
